# Molecular changes in obese and depressive patients are similar to neurodegenerative disorders

**Published:** 2017-10-07

**Authors:** Laleh Habibi, Abbas Tafakhori, Rasoul Hadiani, Maryam Maserat-Mashhadi, Zeinab Kafrash, Shahla Torabi, Mohammad Azhdarzadeh, Seyed Mohammad Akrami, Morteza Mahmoudi, Rasoul Dinarvand

**Affiliations:** 1 Nanotechnology Research Center, School of Pharmacy, Tehran University of Medical Sciences, Tehran, Iran; 2 Department of Medical Genetics, School of Medicine, Tehran University of Medical Sciences, Tehran, Iran; 3 Department of Neurology, School of Medicine, Imam Khomeini Hospital, Tehran University of Medical Sciences, Tehran, Iran; 4 Iranian Center of Neurological Research, Tehran University of Medical Sciences, Tehran, Iran; 5 Heavy Metals Analysis Lab, Food and Drug Laboratories Research Center, Food and Drug Organization, Tehran, Iran; 6 Center of Diabetes Screening, Tehran University of Medical Sciences, Tehran, Iran

**Keywords:** Obesity, Depression, Neurodegeneration, Forkhead Box Protein, Heavy Metals, Lifestyle

## Abstract

**Background:** Neurodegenerative disorders (NDs) are categorized as multifactorial conditions with different molecular and environmental causes. Disturbance of important signaling pathways, such as energy metabolism and inflammation induced by environmental agents, is involved in the pathophysiology of NDs. It has been proposed that changes in the lifestyle and nutrition (metabolism) during mid-life could trigger and accumulate cellular and molecular damages resulting in NDs during aging.

**Methods:** In order to test the hypothesis, we investigated the expression level of two energy metabolism-related [forkhead box O1 (FOXO1) and forkhead box O3 (FOXO3A)] and two pro-inflammatory cytokines [interleukin 1β (IL-1β) and IL-6] genes, using quantitative reverse-transcriptase polymerase chain reaction (qRT-PCR). Furthermore, changes in the ionic concentration of three essential heavy metals [iron (Fe), copper (Cu), and zinc (Zn)] by atomic absorption spectroscopy in patients with NDs, depression, obesity, and diabetes type II, were evaluated and compared with the results of normal individuals.

**Results:** More than half of the participants in obesity, depression, and ND groups had significant up-regulation of FOXO1 and FOXO3A, down-regulation of IL-1β and IL-6, and higher levels of Fe and Cu in their blood. This pattern of gene expression was not repeated in diabetic patients.

**Conclusion:** It could be concluded that individuals affected with different levels of obesity and depression have increased the risk of developing NDs later in life, probably through changes in energy metabolism, inflammatory pathways, and ionic concentrations.

## Introduction

Neurodegenerative disorders (NDs) are a group of diseases resulting from a neuronal loss in different regions of the central nervous system (CNS). It is fully accepted that both environmental and intercellular mechanisms are involved in the pathophysiology of NDs.^1^^,^^2^ Induction of oxidative stress due to increased levels of intercellular metal ions,^3^^,^^4^^,^^5^ mitochondrial dysfunction^6^^,^^7^ disturbances in energy metabolism and autophagy signaling pathways^8^^-^^10^ as well as releasing excessive neuroinflammatory factors in the CNS^11^ are some of the main intercellular pathways involved in neuronal death. Instead, cellular and molecular dysfunctions in neurons are mostly induced by environmental risk factors such as toxic heavy metals,^12^ unregulated homeostasis of essential metal ions^13^ and lifestyle habits.^14^

We have recently suggested that changes in the normal concentration of essential metal ions such as Iron (Fe), Copper (Cu) and Zinc (Zn) rather than other factors could initiate neurodegeneration processes.^15^ Our proposal was mainly based on increasing/decreasing activity of energy metabolism pathways in neurons due to changes in nutritional habits and lifestyle during mid-life. Basically, changes in our habits have resulted in more energy uptake than its consumption, neurological hormone alterations, and changes in the concentration of essential metal ions. All these conditions, which could be reflected as obesity, diabetes type II, and depression at the clinical level, are directly and indirectly connected to defects in energy metabolism pathways, intracytoplasmic metal ion concentrations, and subsequently cell apoptosis.^15^^-^^18^ Changes in lifestyle and nutritional habits have also increased the prevalence of multifactorial diseases such as diabetes type II, obesity, and depression along with NDs.^19^^-^^22^ Moreover, researches have shown that individuals affected by obesity, diabetes type II, and depression have higher risk of developing neurodegeneration during their life.^23^^-^^26^

Forkhead box O1 (FOXO1) and forkhead box O3 (FOXO3A) are two important transcription factors that have major roles in promoting autophagy,^27^^-^^31^ energy metabolism, and stress responses.^32^^-^^42^ Changes in the expression of these genes in CNS could result in neurodegeneration.^9^ Additionally, elevated levels of inflammatory cytokines and alterations in the concentration of essential heavy metals have been reported in affected tissues of NDs, obesity, and diabetes type II.^11^^,^^43^^-^^50^ Although the changes in expression of these two genes have been studied in animal models and are also reported separately in patients suffering from obesity, depression, diabetes type II, and NDs, little is known about the simultaneous pattern of these alterations between all mentioned diseases and healthy individuals. 

Therefore, in the present pilot study, we investigated these alterations by measuring the expression of FOXO1 and FOXO3A in combination with two inflammatory genes interleukin 1β (IL-1β) and IL-6 in patients with diabetes type II, obesity, depression, and NDs, and compared them with normal individuals. We also evaluated alterations of the concentration of three metal ions (Fe, Cu, and Zn) in the proposed groups of patients.

## Materials and Methods

Totally, 105 blood samples were collected from patients diagnosed with ND [mild cognitive impairment (MCI), non-familial Alzheimer's and Parkinson's diseases, n = 26), depression (n = 17), obesity (n = 20) and diabetes type II (n = 21). Twenty-one normal individuals with normal body mass index (BMI), fast blood sugar (FBS) and without any neurological complications were analyzed as a control. Samples with a positive family history of diabetes type II, obesity, depressive and NDs were excluded from the control group. Informed consent was obtained from all individual participants included in the study. Consent form has been approved by Ethical Committee of Iran National Science Foundation. General information of patients in different groups has been indicated in table 1.

ND and depression samples were obtained from Imam Khomeini hospital, department of neurology, Tehran University of Medical Sciences, Iran. Obesity, diabetes type II and normal samples were collected from the Center of Diabetes Screening, Tehran University of Medical Sciences. 

**Table 1:** General information of individuals participated in this study

**Group**	**Sex**		**Age (year) (mean ± SD)**
	**Man [n (%)]**	**Woman [n (%)]**	
Normal (n = 21)	11 (52)	10 (48)	35 ± 3
ND (n = 26)	11 (42)	15 (58)	67 ± 7
Depression (n = 17)	2 (12)	15 (88)	42 ± 5
Obesity (n = 20)	8 (40)	12 (60)	48 ± 2
Diabetes type II (n = 21)	8 (38)	13 (62)	54 ± 2

SD: Standard deviation; ND: Neurodegenerative disorders [including Alzheimer's disease, Parkinson's disease and MCI (mild cognitive impairment)]

Total RNA was extracted from whole blood using the Trizol-chloroform procedure. Briefly, 600 μl of AccuZol (Bioneer, South Korea) was mixed with 1 ml fresh blood. After shaking, 200 μl chloroform (Merck, Germany) was added to the mix, incubated for 15 minutes on ice and centrifuged at 12000 rpm, 15 minutes at 4 ˚C. The clear supernatant was mixed with 500 μl isopropanol (Merck, Germany), incubated 10 minutes on ice and centrifuged at 11000 rpm, 10 minutes, at 4 ˚C. The RNA pellet was then washed 2 times with 70% ethanol at 7000 rpm, 5 minutes, at 4 ˚C and dissolved in 20 μl RNase free ddH_2_O. The quality of RNA was checked on an agarose gel. The quantity and purity of samples were measured using Nano-drop (Thermo scientific, USA) and A260/280 ratio, respectively. High-quality RNA samples were used for cDNA synthesis.

In order to synthesize cDNA, 500 ng of DNase I-treated (Takara, Japan) RNA was mixed with 1 unit AccuPower® CycleScript reverse transcriptase, 1x reaction buffer, 10 mM dNTPs, 0.5 mM oligo dT and 0.5 mM random hexamer primers, RNase inhibitor and up to 20 μl RNase-free ddH_2_O. The mix was incubated at 25 ˚C for 30 seconds for 1 round, and 45 ˚C for 4 minutes and 55 ˚C for 30 seconds, for 12 rounds. The reaction was then heat-inactivated at 95 ˚C for 5 minutes.

Quantitative reverse-transcriptase polymerase chain reaction (qRT-PCR) was performed to quantify the expression of FOXO1, FOXO3A, IL-1B, and IL-6. Glyceraldehyde 3-phosphate dehydrogenase (GAPDH) gene expression was measured as an internal control of the experiment. Primer sequences have been indicated in table 2.

The qRT-PCR reaction was contained 500 ng of cDNA mixed with 25 μl of SYBR green master mix (GeneON, Germany), 300 nM of each reverse and forward primers and up to 50 μl RNase/DNase-free ddH_2_O. PCR reactions were performed in Corbet research instrument (Rotor-Gene^TM^ 6000, Australia) under the following condition: the one-time initial denaturation at 95 ˚C for 10 minutes, and 95 ˚C for 20 seconds, 57 ˚C for 45 seconds, repeated for 40 cycles and followed by melting curve step.

Serums obtained from 3 ml blood samples were used to measure the concentration of free metal ions including Fe, Cu and Zn. Sample dilution for Cu was 1:50 (serum:ddH_2_O), for Fe and Zn was 1:10 (serum:ddH_2_O). Deionized water was used for dilution of samples and standard preparations were prepared with a resistivity of 18.0 MΩ cm (Elga Labwater, Wycombe, Bucks, UK). Working standard solution was freshly prepared in ddH_2_O in 3 dilutions for each metal: Fe standards (0.5, 1, and 2 mg/l), Cu standards (10, 20, and 40 μg/l), and Zn standards (0.1, 0.2 and 0.4 mg/l).

The elemental determination was done by Varian spectra AA-240FS atomic absorption spectrometer (Varian Australia, Pty Ltd, Mulgrave, Victoria, Australia). Flame atomic absorption was used for detecting Fe and Zn. Furnace atomic absorption was applied to measure Cu. 

Seronorm leve2 (SERO AS, Norway) was used in each step to control the qualification of the instrument. Hemolyzed samples were excluded for this test.

This research was designed as a pilot study. qRT-PCR and atomic absorption spectroscopy experiments for each sample were done as triplicate and duplicate, respectively. qRT-PCR data were analyzed using the 2^-∆∆Ct^ method. All data were then analyzed by SPSS software (version 11.5, SPSS Inc., Chicago, IL, USA). Analysis of variance (ANOVA) and Tukey's test were used for data analyses. P less than 0.050 was considered as significant.

**Table 2:** The sequence of primers used in this study

**Gene name**	**Forward 5’-3’**	**Reverse 5’-3’**	**Reference**
FOXO1	TGGACATGCTCAGCAGACATC	TTGGGTCAGGCGGTTCA	^51^
FOXO3A	ATGTGACATGGAGTCCATCATCC	TGTCCACTTGCTGAGAGCAGAT	^52^
IL-1β	ACAGATGAAGTGCTCCTTCCA	GTCGGAGATTCGTAGCTGGAT	^53^
IL-6	GGTACATCCTCGACGGCATCT	GTGCCTCTTTGCTGCTTTCAC	^54^
GAPDH	TGCACCACCAACTGCTTAGC	GGCATGGACTGTGGTCATGAG	^55^

FOXO1: Forkhead box O1; FOXO3A: Forkhead box O3; IL-1β: Interleukin 1β; IL-6: Interleukin 6

**Figure 1:**
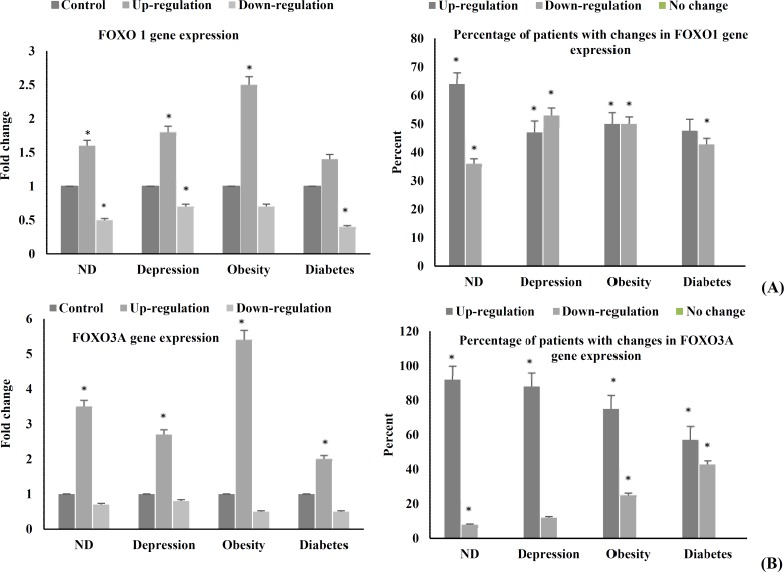
Expression ratio of forkhead genes

The ratio of forkhead box O1 (FOXO1) and forkhead box O3 (FOXO3A) gene expression fold change and percentage of patients in each group of diseases that showed up or down-regulation in these genes are illustrated. Changes in expression of FOXO1 and FOXO3A genes were detected in almost all patients. **A:** More than half of the patients with obesity and depression as well as neurodegenerative diseases (NDs) had significant up-regulation of FOXO1 gene. **B:** The percentage of patients that showed FOXO3A up-regulation is significantly higher in obesity, depression and NDs groups. Patients’ data have been normalized with mean C_t_ values of healthy individuals. The fold change of FOXO1 and FOXO3A for healthy individuals is calculated as 1. The graphs present the fold change and percentage of patients (Y-axis) in each group of diseases (X-axis). Dark gray bars remark expression level in controls, grays bars show up-regulation in patients and light gray bars show down-regulation in patients. In right hand graphs; dark gray bars remark up-regulation and gray bars show down regulation. Standard error of mean (SEM) has been calculated for each bar.

^*^P < 0.050, ND: Neurodegenerative disorders

## Results

***Expression of forkhead transcription factors: energy metabolism pathway:*** In this study, we quantified the expression of FOXO1 and FOXO3A genes in whole blood cells of four groups of patients with NDs, obesity, diabetes type II, and depression and compared them with the normal population. Our data showed that FOXO1 expression was significantly up-regulated (P < 0.050) in more than 50% of obese, depressive, and NDs patients. The number of diabetic patients that showed up-regulation of this gene was not significant. However, the percentage of patients showing down-regulation of FOXO1 was significant (P < 0.011) in all groups of disease (Figure 1A). No significant difference in the pattern of FOXO1 gene expression could be seen between obesity, depression and diabetes type II compared to NDs. 

FOXO3A expression was up-regulated in more than 50% of the patients in all four groups of disease. This change was significant for obesity, depression, and NDs (P < 0.050). Down-regulation of this gene which could be seen in less than 42% of the patients was significant (P < 0.003) for obese, diabetic, and neurodegenerative groups (Figure 1B). Similar to FOXO1, the pattern of FOXO3A gene expression was not significantly different between obese, depressive, and diabetic patients versus patients with NDs.

**Figure 2:**
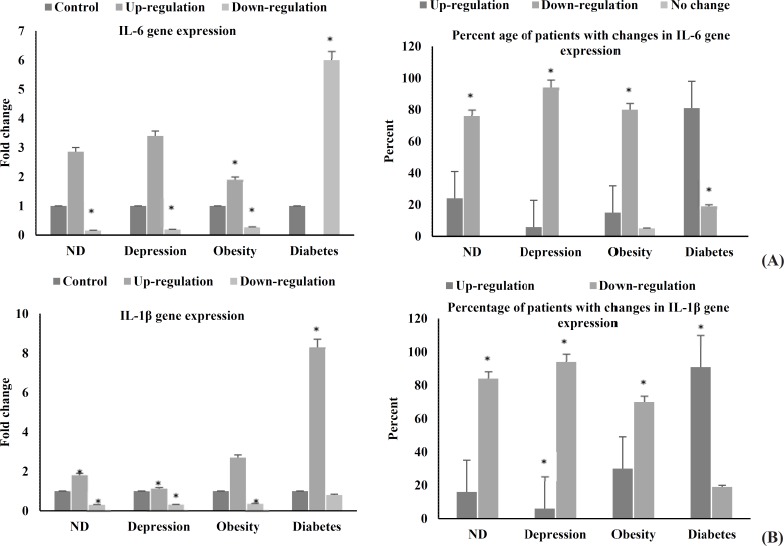
Expression ratio of inflammatory genes

The ratio of interleukin-1β (IL-1β) and interleukin-6 (IL-6) gene expression changes and percentage of patients in each group of diseases that showed up or down-regulation of these genes is illustrated. More than 70% of individuals affected with obesity and depression as well as neurodegenerative diseases (NDs) showed down-regulation of IL-1β (A) and IL-6 (B). Up-regulation of IL-1β and IL-6 in more than 80% of diabetic patients has been detected in this study. Fold change (gene expression) have been normalized with mean C_t_ values of healthy individuals. The fold change of IL-1β and IL-6 for healthy individuals was calculated as 1. The graphs present the fold change and percentages of patients (Y-axis) in each group of diseases (X-axis). In left hand graphs; Dark gray bars remark expression level in controls, grays bars show up-regulation in patients and light gray bars show down-regulation in patients. In right hand graphs; dark gray bars remark up-regulation, gray bars show down-regulation, and light gray bars show no change. Standard error of the mean (SEM) has been calculated for each bar.

^*^P < 0.001, ND: Neurodegenerative; IL: Interleukin

There was no significant correlation between changes in the expression of FOXO1 / FOXO3A and clinical data such as BMI, FBS, low-density lipoprotein (LDL), high-density lipoprotein (HDL), cholesterol, and triglyceride in this study.

***Expression of inflammatory factors:*** In order to check the inflammatory status of our patients, we analyzed the RNA expression of IL-1β and IL-6 (two prominent inflammatory cytokines) in whole blood samples. The results revealed that more than 70% of the patients suffering from NDs, depression, and obesity, had decreased expression of both IL-1β and IL-6 (P < 0.001). However, more than 80% of the diabetic participants showed overexpression of these genes in their blood (Figure 2, A and B). 

There was no significant correlation between changes in the expression of IL-1β / IL-6 and clinical data including BMI, FBS, LDL, HDL, cholesterol, and triglyceride in this study. 

**Figure 3:**
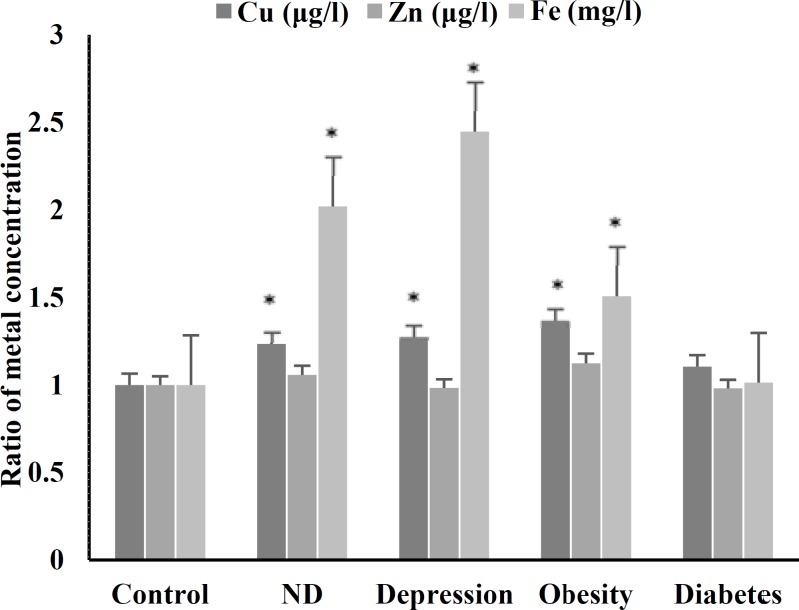
Mean ratio levels of free metal ions in serum of the patients

***Determination of essential heavy metal concentration:*** Measurement of free Fe, Cu, and Zn ions in the serum of patients, as compared with healthy individuals, revealed that the concentration of Fe and Cu increased significantly (P < 0.050) in patients with obesity, depression, and NDs. These changes were not significant between diabetic patients and healthy participants (Figure 3). Changes in the free Zn ion were not significant, as well.

The mean concentration of free iron (Fe) and copper (Cu) in the serum of patients affected with obesity, depression and neurodegenerative diseases (NDs) is significantly higher than the control group. The concentration of zinc (Zn) ions shows no significant change in patients groups. Diabetic patients do not show any change in concentrations of essential metal ions. The graphs present average of metal iron concentration in patients and controls (Y-axis) in each group of study (X-axis). Dark gray bars remark serum level of Cu (µg/l), gray bars present Zn (µg/l) levels and light gray bars show Fe (mg/l) levels. Standard error of the mean (SEM) has been calculated for each bar.

^*^P < 0.050, ND: Neurodegenerative disorders

Although Fe and Cu changes in diabetic patients were not significant, we found a significant (P < 0.001) positive correlation between changes in Fe/Cu concentration and FOXO3A gene expression (correlation coefficient of 0.704 and 0.613, respectively) in this group.

A positive correlation (P < 0.050, correlation coefficient of 0.457) was also found between the concentration of Cu and FOXO3A gene expression in obese patients.

## Discussion

In order to test our hypothesis about the relationship between changes in expression of energy metabolism (autophagy) genes and heavy metal concentrations in diabetic, obese, depressive patients and increasing risk of NDs at clinical level, we compared RNA expression of four genes involved in energy metabolism and inflammatory systems as well as determination of essential metal concentrations in blood of two metabolic syndromes (obesity and diabetes type II), an anxiety condition (depression), three NDs (Alzheimer's and Parkinson's diseases and MCI), and normal group. Obesity, diabetes type II and depression are considered as cases with nutritional and lifestyle problems. 

Our data showed that more than half of obese and depressed patients had higher levels of FOXO1 and FOXO3A expression (involved in energy metabolism and autophagy signaling pathways),^27^^-^^33^ lower levels of IL-1β and IL-6 expression (involved in inflammatory responses) and increased concentrations of free Fe and Cu in their serum, as compared to normal individuals. Interestingly, these results were also repeated in cases with NDs. However, the results were not the same for diabetic patients.

Different studies have highlighted the role of FOXO transcription factors in induction of autophagy in various types of cells.^27^^-^^31^ Up-regulation of FOXO1 and FOXO3A in the blood (mainly lymphocytes) of patients with obesity and depression might reflect similar changes in the expression pattern of these genes in neural cells.^56^ Therefore, increasing forkhead gene expression could cause uncontrolled autophagy events, lead to neural death and increase the risk of neurodegeneration in these patients.

Although increased levels of IL-1β and IL-6 in patients with diabetes type II have also been confirmed in different studies,^57^^,^^58^ down-regulation of these two pro-inflammatory genes in obesity, depression, and ND groups could be controversial because most studies have highlighted higher levels of inflammatory cytokines in all mentioned disorders.^47^^,^^59^^,^^60^ Thus, our results could be explained based on the cells we analyzed in this study and the protective roles of IL-1β and IL-6 in different tissues, especially CNS. Most studies reporting elevated levels of inflammatory cytokines have worked specifically on affected tissues of patients with obesity and neurological problems or have searched for the protein in the serum or cerebrospinal fluid (CSF) of these patients.^61^^-^^63^ However, in this study, we focused on the expression of cytokine genes in the blood cells that might respond differently to disease conditions as compared to affected tissues. Additionally, some studies have indicated that IL-1β and IL-6 expression have neuroprotective roles,^64^^-^^66^ and therefore a decrease in their expression below the normal levels could result in neural injury and apoptosis. Because of the multifactorial nature of the diseases studied in this research, it is possible that the same disease in different individuals is resulted from the impairment of different signaling pathways or environmental factors. That is why we also observed higher levels of inflammatory factors and lower levels of energy metabolism genes in fewer cases of obesity, depression, and NDs. 

Another hallmark of our study is the age of our participants. In this research, most of the patients with obesity, depression, and diabetes type II as well as normal individuals were below 50 years old and in the mid-life stage, while the mean age of our ND patients was 67 years. Therefore, the similar pattern of molecular changes in obesity, depressive and NDs group (with different ages) in this study might indicate that disturbance of different signaling pathways and also ionic concentrations during mid-life could increase the rate of NDs during old age.

To the best of our knowledge, this is the first report directly evaluated FOXO1 and FOXO3A expression along with inflammatory gene expression and metal analysis in the serum of obesity, depression, diabetic type II and NDs simultaneously. In conclusion, we propose that people affected with obesity and depression might have a higher risk of developing NDs mostly through changes in the energy metabolism system and essential metal concentrations. Diabetic patients might be at risk of neuronal loss through inflammatory pathways. Another important point is that obesity, depression, and diabetic conditions in early stages or mild forms are mostly ignored either by affected people or clinicians, while changes in the molecular pathways or ionic concentrations and the consequent cellular damages have already started in their body. Thus, informing negligent people could help them to change their lifestyle and start protective measures.

## Notes:

**How to cite this article:** Habibi L, Tafakhori A, Hadiani R, Maserat-Mashhadi M, Kafrash Z, Torabi S, et al. Molecular changes in obese and depressive patients are similar to neurodegenerative disorders. Iran J Neurol 2017; 16(4): 192-200.

## References

[1] Sheikh S, Safia, Haque E, Mir SS (2013). Neurodegenerative diseases: Multifactorial conformational diseases and their therapeutic interventions. J Neurodegener Dis.

[2] Crews L, Masliah E (2010). Molecular mechanisms of neurodegeneration in Alzheimer's disease. Hum Mol Genet.

[3] Nakamura T, Lipton SA (2009). Cell death: Protein misfolding and neurodegenerative diseases. Apoptosis.

[4] Jomova K, Vondrakova D, Lawson M, Valko M (2010). Metals, oxidative stress and neurodegenerative disorders. Mol Cell Biochem.

[5] Melo A, Monteiro L, Lima RM, Oliveira DM, Cerqueira MD, El-Bacha RS (2011). Oxidative stress in neurodegenerative diseases: Mechanisms and therapeutic perspectives. Oxid Med Cell Longev.

[6] Johri A, Beal MF (2012). Mitochondrial dysfunction in neurodegenerative diseases. J Pharmacol Exp Ther.

[7] Hroudova J, Singh N, Fisar Z (2014). Mitochondrial dysfunctions in neurodegenerative diseases: Relevance to Alzheimer's disease. Biomed Res Int.

[8] Blandini F, Braunewell KH, Manahan-Vaughan D, Orzi F, Sarti P (2004). Neurodegeneration and energy metabolism: From chemistry to clinics. Cell Death Differ.

[9] Maiese K (2015). FoxO proteins in the nervous system. Anal Cell Pathol (Amst).

[10] Ghavami S, Shojaei S, Yeganeh B, Ande SR, Jangamreddy JR, Mehrpour M (2014). Autophagy and apoptosis dysfunction in neurodegenerative disorders. Prog Neurobiol.

[11] Amor S, Peferoen LA, Vogel DY, Breur M, van der V, Baker D (2014). Inflammation in neurodegenerative diseases--an update. Immunology.

[12] Chin-Chan M, Navarro-Yepes J, Quintanilla-Vega B (2015). Environmental pollutants as risk factors for neurodegenerative disorders: Alzheimer and Parkinson diseases. Front Cell Neurosci.

[13] Kozlowski H, Janicka-Klos A, Brasun J, Gaggelli E, Valensin D, Valensin G (2009). Copper, iron, and zinc ions homeostasis and their role in neurodegenerative disorders (metal uptake, transport, distribution and regulation). Coordination Chemistry Reviews.

[14] Brown RC, Lockwood AH, Sonawane BR (2005). Neurodegenerative diseases: An overview of environmental risk factors. Environ Health Perspect.

[15] ManafiRad A, Farzadfar F, Habibi L, Azhdarzadeh M, Aghaverdi H, Tehrani KH (2014). Is amyloid-beta an innocent bystander and marker in Alzheimer's disease? Is the liability of multivalent cation homeostasis and its influence on amyloid-beta function the real mechanism?. J Alzheimers Dis.

[16] Lavallard VJ, Meijer AJ, Codogno P, Gual P (2012). Autophagy, signaling and obesity. Pharmacol Res.

[17] Muriach M, Flores-Bellver M, Romero FJ, Barcia JM (2014). Diabetes and the brain: Oxidative stress, inflammation, and autophagy. Oxid Med Cell Longev.

[18] Stuart MJ, Baune BT (2012). Depression and type 2 diabetes: Inflammatory mechanisms of a psychoneuroendocrine co-morbidity. Neurosci Biobehav Rev.

[19] Nguyen DM, El-Serag HB (2010). The epidemiology of obesity. Gastroenterol Clin North Am.

[20] Cheng D (2005). Prevalence, predisposition and prevention of type II diabetes. Nutr Metab (Lond ).

[21] Hidaka BH (2012). Depression as a disease of modernity: Explanations for increasing prevalence. J Affect Disord.

[22] Dorsey ER, George BP, Leff B, Willis AW (2013). The coming crisis: Obtaining care for the growing burden of neurodegenerative conditions. Neurology.

[23] Ashrafian H, Harling L, Darzi A, Athanasiou T (2013). Neurodegenerative disease and obesity: What is the role of weight loss and bariatric interventions?. Metab Brain Dis.

[24] Janson J, Laedtke T, Parisi JE, O'Brien P, Petersen RC, Butler PC (2004). Increased risk of type 2 diabetes in Alzheimer disease. Diabetes.

[25] Wuwongse S, Chang RC, Law AC (2010). The putative neurodegenerative links between depression and Alzheimer's disease. Prog Neurobiol.

[26] Caraci F, Copani A, Nicoletti F, Drago F (2010). Depression and Alzheimer's disease: Neurobiological links and common pharmacological targets. Eur J Pharmacol.

[27] Shin HR, Kim H, Kim KI, Baek SH (2016). Epigenetic and transcriptional regulation of autophagy. Autophagy.

[28] Xu P, Das M, Reilly J, Davis RJ (2011). JNK regulates FoxO-dependent autophagy in neurons. Genes Dev.

[29] Zhou J, Liao W, Yang J, Ma K, Li X, Wang Y (2012). FOXO3 induces FOXO1-dependent autophagy by activating the AKT1 signaling pathway. Autophagy.

[30] Wang S, Xia P, Huang G, Zhu P, Liu J, Ye B (2016). FoxO1-mediated autophagy is required for NK cell development and innate immunity. Nat Commun.

[31] Sengupta A, Molkentin JD, Yutzey KE (2009). FoxO transcription factors promote autophagy in cardiomyocytes. J Biol Chem.

[32] Kousteni S (2012). FoxO1, the transcriptional chief of staff of energy metabolism. Bone.

[33] Nakae J, Oki M, Cao Y (2008). The FoxO transcription factors and metabolic regulation. FEBS Lett.

[34] Brunet A, Sweeney LB, Sturgill JF, Chua KF, Greer PL, Lin Y (2004). Stress-dependent regulation of FOXO transcription factors by the SIRT1 deacetylase. Science.

[35] Tran H, Brunet A, Grenier JM, Datta SR, Fornace AJ Jr, DiStefano PS (2002). DNA repair pathway stimulated by the forkhead transcription factor FOXO3a through the Gadd45 protein. Science.

[36] Warr MR, Binnewies M, Flach J, Reynaud D, Garg T, Malhotra R (2013). FOXO3A directs a protective autophagy program in haematopoietic stem cells. Nature.

[37] Zhao Y, Yang J, Liao W, Liu X, Zhang H, Wang S (2010). Cytosolic FoxO1 is essential for the induction of autophagy and tumour suppressor activity. Nat Cell Biol.

[38] Samuel VT, Choi CS, Phillips TG, Romanelli AJ, Geisler JG, Bhanot S (2006). Targeting foxo1 in mice using antisense oligonucleotide improves hepatic and peripheral insulin action. Diabetes.

[39] Relling DP, Esberg LB, Fang CX, Johnson WT, Murphy EJ, Carlson EC (2006). High-fat diet-induced juvenile obesity leads to cardiomyocyte dysfunction and upregulation of Foxo3a transcription factor independent of lipotoxicity and apoptosis. J Hypertens.

[40] Yang XF, Fang P, Meng S, Jan M, Xiong X, Yin Y (2009). The FOX transcription factors regulate vascular pathology, diabetes and Tregs. Front Biosci (Schol Ed).

[41] Zemva J, Schilbach K, Stohr O, Moll L, Franko A, Krone W (2012). Central FoxO3a and FoxO6 expression is down-regulated in obesity induced diabetes but not in aging. Exp Clin Endocrinol Diabetes.

[42] Guidotti G, Calabrese F, Anacker C, Racagni G, Pariante CM, Riva MA (2013). Glucocorticoid receptor and FKBP5 expression is altered following exposure to chronic stress: Modulation by antidepressant treatment. Neuropsychopharmacology.

[43] Gregor MF, Hotamisligil GS (2011). Inflammatory mechanisms in obesity. Annu Rev Immunol.

[44] Donath MY, Shoelson SE (2011). Type 2 diabetes as an inflammatory disease. Nat Rev Immunol.

[45] Slavich GM, Irwin MR (2014). From stress to inflammation and major depressive disorder: A social signal transduction theory of depression. Psychol Bull.

[46] Ramesh G, MacLean AG, Philipp MT (2013). Cytokines and chemokines at the crossroads of neuroinflammation, neurodegeneration, and neuropathic pain. Mediators Inflamm.

[47] Fain JN (2006). Release of interleukins and other inflammatory cytokines by human adipose tissue is enhanced in obesity and primarily due to the nonfat cells. Vitam Horm.

[48] Spranger J, Kroke A, Mohlig M, Hoffmann K, Bergmann MM, Ristow M (2003). Inflammatory cytokines and the risk to develop type 2 diabetes: Results of the prospective population-based European Prospective Investigation into Cancer and Nutrition (EPIC)-Potsdam Study. Diabetes.

[49] Kozlowski H, Luczkowski M, Remelli M, Valensin D (2012). Copper, zinc and iron in neurodegenerative diseases (Alzheimer's, Parkinson's and prion diseases). Coordination Chemistry Reviews.

[50] Thomas B, Gautam A, Prasad BR, Kumari S (2013). Evaluation of micronutrient (zinc, copper and iron) levels in periodontitis patients with and without diabetes mellitus type 2: A biochemical study. Indian J Dent Res.

[51] Costa CS, Rohden F, Hammes TO, Margis R, Bortolotto JW, Padoin AV (2011). Resveratrol upregulated SIRT1, FOXO1, and adiponectin and downregulated PPARgamma1-3 mRNA expression in human visceral adipocytes. Obes Surg.

[52] Qiu H, Wang F, Liu C, Xu X, Liu B (2011). TEAD1-dependent expression of the FoxO3a gene in mouse skeletal muscle. BMC Mol Biol.

[53] Li J, Moran T, Swanson E, Julian C, Harris J, Bonen DK (2004). Regulation of IL-8 and IL-1beta expression in Crohn's disease associated NOD2/CARD15 mutations. Hum Mol Genet.

[54] Oberbach A, Schlichting N, Bluher M, Kovacs P, Till H, Stolzenburg JU (2010). Palmitate induced IL-6 and MCP-1 expression in human bladder smooth muscle cells provides a link between diabetes and urinary tract infections. PLoS One.

[55] Habibi L, Shokrgozar MA, Tabrizi M, Modarressi MH, Akrami SM (2014). Mercury specifically induces LINE-1 activity in a human neuroblastoma cell line. Mutat Res Genet Toxicol Environ Mutagen.

[56] Habibi L, Ebtekar M, Jameie SB (2009). Immune and nervous systems share molecular and functional similarities: Memory storage mechanism. Scand J Immunol.

[57] de la Monte SM, Longato L, Tong M, Wands JR (2009). Insulin resistance and neurodegeneration: roles of obesity, type 2 diabetes mellitus and non-alcoholic steatohepatitis. Curr Opin Investig Drugs.

[58] Pickup JC, Crook MA (1998). Is type II diabetes mellitus a disease of the innate immune system?. Diabetologia.

[59] Stegenga ME, van der Crabben SN, Dessing MC, Pater JM, van den Pangaart PS, de Vos AF (2008). Effect of acute hyperglycaemia and/or hyperinsulinaemia on proinflammatory gene expression, cytokine production and neutrophil function in humans. Diabet Med.

[60] Allan SM (2000). The role of pro- and antiinflammatory cytokines in neurodegeneration. Ann N Y Acad Sci.

[61] Young JJ, Bruno D, Pomara N (2014). A review of the relationship between proinflammatory cytokines and major depressive disorder. J Affect Disord.

[62] Dowlati Y, Herrmann N, Swardfager W, Liu H, Sham L, Reim EK (2010). A meta-analysis of cytokines in major depression. Biol Psychiatry.

[63] Smith JA, Das A, Ray SK, Banik NL (2012). Role of pro-inflammatory cytokines released from microglia in neurodegenerative diseases. Brain Res Bull.

[64] Jia JP, Meng R, Sun YX, Sun WJ, Ji XM, Jia LF (2005). Cerebrospinal fluid tau, Abeta1-42 and inflammatory cytokines in patients with Alzheimer's disease and vascular dementia. Neurosci Lett.

[65] Carlson NG, Wieggel WA, Chen J, Bacchi A, Rogers SW, Gahring LC (1999). Inflammatory cytokines IL-1 alpha, IL-1 beta, IL-6, and TNF-alpha impart neuroprotection to an excitotoxin through distinct pathways. J Immunol.

[66] Ali C, Nicole O, Docagne F, Lesne S, MacKenzie ET, Nouvelot A (2000). Ischemia-induced interleukin-6 as a potential endogenous neuroprotective cytokine against NMDA receptor-mediated excitotoxicity in the brain. J Cereb Blood Flow Metab.

